# Memristive TiO_2_: Synthesis, Technologies, and Applications

**DOI:** 10.3389/fchem.2020.00724

**Published:** 2020-10-02

**Authors:** Georgii A. Illarionov, Sofia M. Morozova, Vladimir V. Chrishtop, Mari-Ann Einarsrud, Maxim I. Morozov

**Affiliations:** ^1^Laboratory of Solution Chemistry of Advanced Materials and Technologies, ITMO University, St. Petersburg, Russia; ^2^Department of Material Science and Engineering, NTNU Norwegian University of Science and Technology, Trondheim, Norway

**Keywords:** TiO_2_, memristor, nanoparticles, neuromorphic, electronic oxides

## Abstract

Titanium dioxide (TiO_2_) is one of the most widely used materials in resistive switching applications, including random-access memory, neuromorphic computing, biohybrid interfaces, and sensors. Most of these applications are still at an early stage of development and have technological challenges and a lack of fundamental comprehension. Furthermore, the functional memristive properties of TiO_2_ thin films are heavily dependent on their processing methods, including the synthesis, fabrication, and post-fabrication treatment. Here, we outline and summarize the key milestone achievements, recent advances, and challenges related to the synthesis, technology, and applications of memristive TiO_2_. Following a brief introduction, we provide an overview of the major areas of application of TiO_2_-based memristive devices and discuss their synthesis, fabrication, and post-fabrication processing, as well as their functional properties.

## Introduction

Titanium dioxide (TiO_2_) is a multifunctional semiconductor that exists in three crystalline forms: anatase, rutile, and brookite. Owing to an appropriate combination of physical and chemical properties, environmental compatibility, and low production cost, polycrystalline TiO_2_ has found a large variety of applications and is considered to be a promising material for future technologies. One of the most distinctive physical properties of this material is its high photocatalytic activity (Nam et al., 2019); however, more recently it has attracted growing interest because of its resistive switching abilities (Yang et al., 2008).

The realization of neuromorphic resistive memory in TiO_2_ thin films (Strukov et al., 2008) marked an important milestone in the search for bio-inspired technologies (Chua and Kang, 1976). Many research proposals urged a focus on memristivity as the common feature of two electrical models: (i) electromigration of point defects in titanium oxide systems (Baiatu et al., 1990; Jameson et al., 2007) and (ii) voltage-gated ionic channels in the membranes of biological neurons (Hodgkin and Huxley, 1952). In this regard, memristors functionally mimic the synaptic plasticity of biological neurons, and thus can be implemented in artificial and hybrid neural networks. This includes a new paradigm of future computing systems (Zidan, 2018) and biocompatible electronics such as biointerfaces and biohybrid systems (Chiolerio et al., 2017).

**Figure 1:**
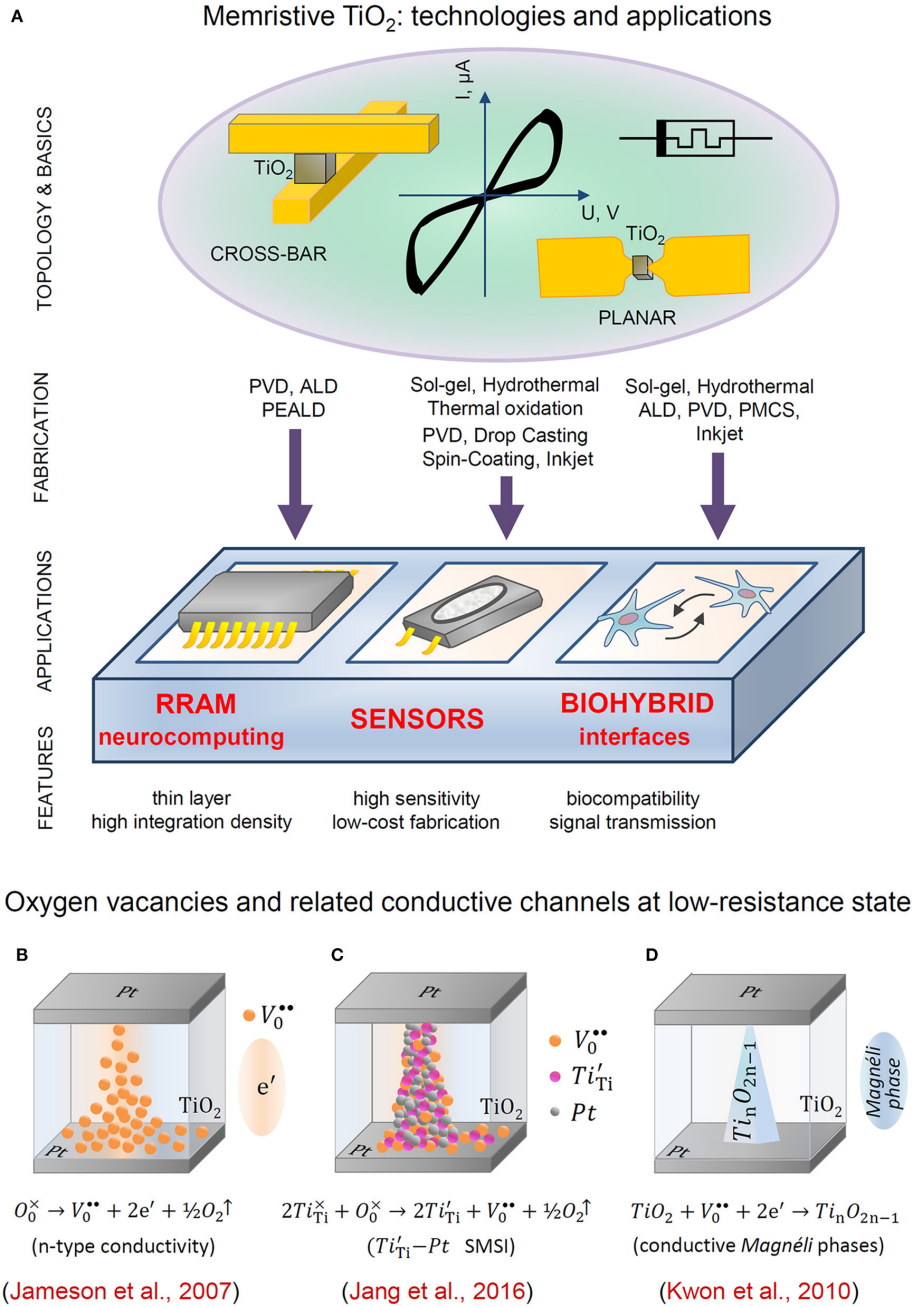
**(A)** Technologies and applications of memristive TiO_2_ thin films. **(B–D)** Formation of the conductive filament: **(B)** electromigration of oxygen vacancies inducing the *n*-type conductivity region; **(C)** detachment and migration of electrode metal atoms due to strong metal support interaction (SMSI); **(D)** formation of a conductive Magnéli phase.

Currently, the development of TiO_2_ memristors is associated with their use in modern highly technological applications, such as resistive random-access memory (RRAM), biohybrid systems, and sensors, as schematically shown in Figure 1A. In this mini-review, we briefly outline and summarize the key milestone achievements, as well as recent advances in the synthesis, fabrication, and application of TiO_2_-based memristors. A special focus is placed on the relationships between the synthesis and deposition methods, the effects of post-synthesis treatment, and the resistive switching properties.

## Oxygen Deficiency and Resistive Switching Mechanisms

The basic scenario of resistive switching in TiO_2_ (Jameson et al., 2007) assumes the formation and electromigration of oxygen vacancies between the electrodes (Baiatu et al., 1990), so that the distribution of concomitant *n*-type conductivity (Janotti et al., 2010) across the volume can eventually be controlled by an external electric bias, as schematically shown in Figure 1B. Direct observations with transmission electron microscopy (TEM) revealed more complex electroforming processes in TiO_2_ thin films. In one of the studies, a continuous Pt filament between the electrodes was observed in a planar Pt/TiO_2_/Pt memristor (Jang et al., 2016). As illustrated in Figure 1C, the corresponding switching mechanism was suggested as the formation of a conductive nanofilament with a high concentration of ionized oxygen vacancies and correspondingly reduced Ti^3+^ ions. These ions induce detachment and migration of Pt atoms from the electrode via strong metal–support interactions (Tauster, 1987). Another TEM investigation of a conductive TiO_2_ nanofilament revealed it to be a Magnéli phase Ti_*n*_O_2n−1_ (Kwon et al., 2010). Supposedly, its formation results from an increase in the concentrations of oxygen vacancies within a local nanoregion above their thermodynamically stable limit. This scenario is schematically shown in Figure 1D. Other hypothesized point defect mechanisms involve a contribution of cation and anion interstitials, although their behavior has been studied more in tantalum oxide (Wedig et al., 2015; Kumar et al., 2016). The plausible origins and mechanisms of memristive switching have been comprehensively reviewed in topical publications devoted to metal oxide memristors (Yang et al., 2008; Waser et al., 2009; Ielmini, 2016) as well as TiO_2_ (Jeong et al., 2011; Szot et al., 2011; Acharyya et al., 2014). The resistive switching mechanisms in memristive materials are regularly revisited and updated in the themed review publications (Sun et al., 2019; Wang et al., 2020).

## Applications

### RRAM and the New Computing Paradigm

As they mimic the synapses in biological neurons, memristors became the key component for designing novel types of computing and information systems based on artificial neural networks, the so-called neuromorphic electronics (Zidan, 2018; Wang and Zhuge, 2019; Zhang et al., 2019b). Electronic artificial neurons with synaptic memristors are capable of emulating the associative memory, an important function of the brain (Pershin and Di Ventra, 2010). In addition, the technological simplicity of thin-film memristors based on transition metal oxides such as TiO2 allows their integration into electronic circuits with extremely high packing density. Memristor crossbars are technologically compatible with traditional integrated circuits, whose integration can be implemented within the complementary metal–oxide–semiconductor platform using nanoimprint lithography (Xia et al., 2009). Nowadays, the size of a Pt-TiOx-HfO2-Pt memristor crossbar can be as small as 2 nm (Pi et al., 2019). Thus, the inherent properties of memristors such as non-volatile resistive memory and synaptic plasticity, along with feasibly high integration density, are at the forefront of the new-type hardware performance of cognitive tasks, such as image recognition (Yao et al., 2017). The current state of the art, prospects, and challenges in the new brain-inspired computing concepts with memristive implementation have been comprehensively reviewed in topical papers (Jeong et al., 2016; Xia and Yang, 2019; Zhang et al., 2020). These reviews postulate that the newly emerging computing paradigm is still in its infancy, while the rapid development and current challenges in this field are related to the technological and materials aspects. The major concerns are the lack of understanding of the microscopic picture and the mechanisms of switching, as well as the unproven reliability of memristor materials. The choice of memristive materials as well as the methods of synthesis and fabrication affect the properties of memristive devices, including the amplitude of resistive switching, endurance, stochasticity, and data retention time.

### Biointerfaces, Biomimicking, and Biohybrid Systems

The neuromorphic nature of the resistive switching in TiO_2_ memristors has triggered a series of studies addressing their functional coupling with living biological systems. The common features of the electroconductive behavior of memristive and biological neural networks have been revised in terms of physical, mathematical, and stochastic models (Chua, 2013; Feali and Ahmadi, 2016). The memristive electronics was shown to support important synaptic functions such as spike timing-dependent plasticity (Jo et al., 2010; Pickett et al., 2013). Recently, a memristive simulation of important biological synaptic functions such as non-linear transmission characteristics, short-/long-term plasticity, and paired-pulse facilitation has been reported for hybrid organic–inorganic memristors using Ti-based maleic acid/TiO_2_ ultrathin films (Liu et al., 2020). In relation to this, functionalized TiO_2_ memristive systems may be in competition with the new generation of two-dimensional memristive materials such as WSe_2_ (Zhu et al., 2018), MoS_2_ (Li et al., 2018), MoS_2_/graphene (Kalita et al., 2019), and other systems (Zhang et al., 2019a) with ionic coupling, ionic modulation effects, or other synapse-mimicking functionalities. Furthermore, the biomimetic fabrication of TiO_2_ (Seisenbaeva et al., 2010; Vijayan and Puglia, 2019; Kumar et al., 2020) opens up new horizons for its versatile microstructural patterning and functionalizations.

The first study addressing the experimental convergence between *in vitro* spiking neurons and spiking memristors was attempted in 2013 (Gater et al., 2013). A few years later, Gupta et al. (2016) used TiO_2_ memristors to compress information on biological neural spikes recorded in real time. In these *in vitro* studies electrical communication with biological cells, as well as their incubation, was investigated using multielectrode arrays (MEAs). Alternatively, TiO_2_ thin films may serve as an interface material in various biohybrid devices. The bio- and neurocompatibility of a TiO_2_ film has been demonstrated in terms of its excellent adsorption of polylysine and primary neuronal cultures, high vitality, and electrophysiological activity (Roncador et al., 2017). Thus, TiO_2_ can be implemented as a nanobiointerface coating and integrated with memristive electronics either as a planar configuration of memristors and electrodes (Illarionov et al., 2019) or as a functionalization of MEAs to provide good cell adhesion and signal transmission. The known examples are electrolyte/TiO_2_/Si(*p*-type) capacitors (Schoen and Fromherz, 2008) or capacitive TiO_2_/Al electrodes (Serb et al., 2020). As a demonstration of the state of the art, an attempt at memristive interlinking between the brain and brain-inspired devices has been recently reported (Serb et al., 2020). The long-term potentiation and depression of TiO_2_-based memristive synapses have been demonstrated in relation to the neuronal firing rates of biologically active cells. Further advancement in this area is expected to result in scalable on-node processors for brain–chip interfaces (Gupta et al., 2016). As of 2017, the state of the art of, and perspectives on, coupling between the resistive switching devices and biological neurons have been reviewed (Chiolerio et al., 2017).

### Sensors

Apart from proximately neuromorphic technologies, TiO_2_-based memristors have also found application in various sensors. The principle of memristive sensorics is based on the dependency of the resistive switching on various external stimuli. This includes recording of mechanical energy (Vilmi et al., 2016), hydrogen detection (Hossein-Babaei and Rahbarpour, 2011; Strungaru et al., 2015; Haidry et al., 2017; Vidiš et al., 2019), γ-ray sensing (Abunahla et al., 2016), and various fluidic-based sensors, such as sensors for pH (Hadis et al., 2015a) and glucose concentration (Hadis et al., 2015b). In addition, TiO_2_ thin films may generate photoinduced electron–hole pairs, which give rise to UV radiation sensors (Hossein-Babaei et al., 2012). Recently, the biosensing properties of TiO_2_-based memristors have been demonstrated in the detection of the bovine serum albumin protein molecule (Sahu and Jammalamadaka, 2019). Furthermore, this work has also demonstrated that the introduction of an additional graphene oxide layer may effectively prevent the growth of multidimensional and random conductive paths, resulting in a lower switching voltage, better endurance, and a higher resistance switching ratio. This opens up a new horizon for further functional convergence of metal oxides and two-dimensional memristive materials and interfaces (Zhang et al., 2019a).

## Synthesis and Fabrication

The functionality of TiO_2_ memristors is largely related to the phase purity, phase structure, crystallinity, and defect structure. In turn, all these parameters depend on the synthesis method, fabrication processing, and post-fabrication treatment (Diebold, 2003; Chen and Mao, 2007; Goren et al., 2014). Most TiO_2_ memristors consist of anatase or rutile because of the stability of these polymorphs. The formation of oxygen vacancies and concomitant *n*-type conductivity can be controlled at temperatures above 300°C (Hou et al., 2018).

The fabrication of a TiO_2_ memristive device typically consists of (i) the synthesis of a nanostructured material, (ii) deposition of the functional layer, (iii) arrangement of the electrodes, and (iv) post-processing annealing at an elevated temperature under a suitable atmosphere. The first stage is required for traditional chemical synthesis routes, while the first two stages take place at the same time for physical deposition methods.

The choice of fabrication route is thus a trade-off in complexity, cost, scalability, desirable topology (film thickness and topological feature size of the electrode areas), threshold electroforming voltage (*V*_T_), retention time, switching time, and the resistive switching ratio (*R*_OFF_/*R*_ON_). The main technological, topological, and exploitative characteristics of typical memristive devices are summarized in Table 1 and will be reviewed in the following sections.

**Table 1:** Overview of the structure and electrical properties of TiO_2_-based memristors obtained by various synthesis methods.

**Synthesis method**	**Deposition method (annealing temperature, time, and atmosphere)**	**Structure**	Feature size^*^, μm^**2**^ (TiO_**2**_ phase)	**Thickness, nm (particle size, nm)**	**$V_{T}^{**}$, V**	***R*_**OFF**_/*R*_**ON**_**	**Application**	**Retention time, s (switching time, s)**	**Reference**
Sol–gel	Spin coating (550°C, 10 h, air)	Cellular, Al/TiO_2_/FTO	2 × 10^6^ (anatase)	35	3.9	2 × 10^5^	General	10^4^ (N/D)	(Tao et al., 2020)
Sol–gel	Inkjet (200°C, 2 h, Air)	Planar, Au/TiO_2_/Au	3 (anatase)	400 (7^***^)	~4	~20	Cell biology	N/D	(Illarionov et al., 2019)
Sol–gel	Drop casting (N/D)	Crossbar, Ag/TiO_2_/Cu	4 × 10^6^ (amorphous)	4.5 × 10^4^	0.5	10^7^	γ-ray sensor	4 × 10^4^ (50 ÷ 360)	(Abunahla et al., 2018)
Sol–gel	Inkjet (150°C, 15 min, N_2_)	Cellular, Ag/TiO_2_/Ag/PET	3,600	10–160	~1.5	500	Mechanical sensor	N/D	(Vilmi et al., 2016)
Sol–gel	Spin coating (500°C, 1 h, air)	Cells array, Al/TiO_2_/FTO	2 × 10^5^ (anatase)	100	~1.8	>300	RRAM	10^4^ (10)	(Hu et al., 2020)
Hydrothermal	Dip coating (450°C, 2 h, air)	Sandwiched, Ag/TiO_2_/Al	N/D (anatase)	265	0.7	100	RRAM	N/D	(Dongale et al., 2014)
Hydrothermal	Dip coating (500°C, 3 h, air)	Sandwiched, Al/TiO_2_/Ti	7.9 × 10^5^ (anatase nanowires)	N/D	~3	70	RRAM	10^4^ (N/D)	(Xiao et al., 2017)
Hydrothermal	Dip coating (300°C, 2 h, air)	Sandwiched, Ag/TiO_2_/FTO	1.3 × 10^7^ (rutile + anatase, rutile)	7,000	1.2	>10	RRAM	4 × 10^6^ (N/D)	(Irshad et al., 2019)
Solid state, 2D colloid	Dip coating	Crossbar, Al/TiO_2_/Pt/Ti/SiO_2_/Si	4	2	0.5 ÷ 1.5	10^6^	RRAM	10^4^ (20 × 10^−9^)	(Dai et al., 2017)
Thermal oxidation	–	Sandwiched, Ir/TiO_2_/TiN	N/D	4	>1.5 (set)	~100	RRAM	10^4^ (10^−7^)	(Park et al., 2011)
Thermal oxidation	– (650°C, 1 h, air)	Cellular, Ti/TiO_2_/Ti	~3 × 10^6^ (rutile)	400 (50)^****^	2	~4	Humidity sensor	6 × 10^5^ (~10^−2^)	(Hossein-Babaei and Alaei-Sheini, 2016)
Anodizing	–	Cellular, Cu/TiO_2_/Ti	4	8, 11, 29	−1.5	<80	RRAM	N/D	(Aglieri et al., 2018)
Anodizing	– (550°C, 1 h, N_2_/H_2_ 24/1)	Cells array, Pt/TiO_2_/Ti	~10^8^	<100	<1	~56	General	N/D	(Miller et al., 2010)
PVD, ALD	–	Crossbar, Pt/TiO_2_/HfO_2_/Pt	4 × 10^−6^	7	~1.8	450	RRAM, computing	120 (N/D)	(Pi et al., 2019)
PEALD	–	Crossbar, Al/TiO_2_/Al	3,600 (amorphous)	13	2.1	>100	RRAM	N/D	(Jeong et al., 2010b)
PEALD	–	Crossbar, Pt/Ni/TiO_2_/Al_2_O_3_/Pt	4,900 (amorphous)	12	~0.5 ÷ 1.5	~100	RRAM	N/D	(Jeong et al., 2010a)
PVD/RMS	– (600, 800°C, 3 h, air)	Crossbar, Pt/TiO_2_/Pt	N/D (anatase)	2,000 (40–50)^***^	~0.2 ÷ 1.0	<6 × 10^5^	H_2_ sensor	N/D (5)	(Haidry et al., 2017)
PVD/RFS	– (400°C, 15 min, N_2_)	Crossbar, Ni/TiO_2_/Ni	1.1 (anatase)	10	~0.8	10^4^	RRAM	N/D	(Cortese et al., 2016)
PVD/RS	–	Cellular, Al/TiO_2_/Au	100	50	0.5	12	General	N/D (10^−3^)	(Ghenzi and Levy, 2018)
PVD/RS	–	Crossbar, Pt/TiO_2_/Pt/Cr	2.25, 9 (amorphous and anatase)	30 (10)^***^	~0.5	~100	RRAM	N/D	(Strachan et al., 2013)
PMCS	–	Planar, glass/TiO_2_/Al	1.77 × 10^8^ (rutile and anatase)	30	N/D	N/D	Bio-interface	N/D	(Roncador et al., 2017)
PLD	–	Cellular, Cu/TiO_2_/Pt	1.26 × 10^5^	100	~0.2	~3 × 10^3^	RRAM	100 (250 × 10^−9^)	(Sahu et al., 2020)

*~, estimated or recalculated values*;

* *electrode area*;

** *threshold voltage*;

*** *by Scherrer equation*;

**** *by SEM, scanning electron microscopy*.

*2D, two-dimensional; ALD, atomic layer deposition; MRS, magnetron reactive sputtering; PEALD, plasma-enhanced atomic layer deposition; PVD, physical vapor deposition; RFS, radio frequency sputtering; R_OFF_/R_ON_, resistive switching ratio; RRAM, resistive random-access memory; RS, reactive sputtering; V_T_, threshold electroforming voltage*.

### Synthesis

#### Chemical Approaches

##### Sol–gel

In this process, a liquid solution is converted into a viscoelastic gel phase. In the classical concept of sol–gel, the phase purity, size, and shape of synthesized TiO_2_ nanoparticles are considered in relation to the hydrolysis and condensation reactions, the reactivity and concentration of the precursor (titanium alkoxide or TiCl_4_), the solvent type, and the temperature (Cargnello et al., 2014). In addition, the early-stage processes such as nucleation, crystal growth, and aggregation (Teychené et al., 2020) may play a crucial role in the sol–gel synthesis of TiO_2_ nanoparticles (Cheng et al., 2017). The memristive devices fabricated using the sol–gel method have various areas of application and operate at a threshold voltage ranging from ~0.5 V (Abunahla et al., 2018) to 1.5 V (Vilmi et al., 2016; Hu et al., 2020) or higher (Illarionov et al., 2019) with a resistive switching ratio *R*_OFF_/*R*_ON_ of 10^1^-10^5^ (Table 1). Thus, the functional parameters of these devices may be variable with respect to the morphology and purity of the sol–gel product, deposition method (see section Fabrication), and annealing conditions (see section Annealing and Electric Properties).

##### Thermal oxidation

Polycrystalline TiO_2_ typically in the form of rutile can be obtained by thermal oxidation of a titanium layer at temperatures in the range 500–800°C (Cao et al., 2009). This method allows fabrication of TiO_2_ films with thicknesses down to 4 nm (Park et al., 2011). Furthermore, thermal oxidation is a cost-efficient method that is compatible with standard RRAM manufacturing technology (Acharyya et al., 2014). However, processing temperatures above 500°C might cause the formation of crystallographic line defects or microcracks.

##### Hydrothermal synthesis

This is defined as heterogeneous reactions in aqueous media under high pressure and temperature sufficient to dissolve and recrystallize materials that are insoluble in water under normal conditions (Byrappa and Yoshimura, 2001). Various metal alkoxides (Ti(OR)_4_, R = C_2_H_5_, i-C_3_H_7_, C_4_H_9_) or TiCl_4_ have been used as precursors (Oh et al., 2009; Zhang et al., 2011; Senthilkumar et al., 2013; Dongale et al., 2014; Irshad et al., 2019). In this process, temperatures up to 230°C and high pressures (around 200 bar) facilitate the formation of a crystalline product at relatively low temperatures (Dalod et al., 2017). The TiO_2_-based materials obtained by this method show reasonable *R*_OFF_/*R*_ON_ switching ratios of up to 100 (Dongale et al., 2014; Xiao et al., 2017), although higher values (>10^4^) have also been reported (Senthilkumar et al., 2013). Additional control over the size and shape of TiO_2_ particles can be achieved by means of a solvothermal approach (Dinh et al., 2009). Recently, this method has been successfully applied to obtain sub-10 nm TiO_2_ nanoparticles that are capable of forming self-assembled monolayers and that possess resistive switching properties (Schmidt et al., 2017).

##### Electrochemical oxidation

Using an electrochemical method or anodizing, the oxidation of a titanium foil in an electrochemical cell provides nanostructures of TiO_2_ (Yoo et al., 2013). With this method, the composition, thickness, and structure can be controlled by choosing an appropriate substrate, electrolyte, and electrochemical conditions. Only a few studies have addressed this method of fabricating TiO_2_-based memristors (Miller et al., 2010; Yoo et al., 2013; Aglieri et al., 2018; Zaffora et al., 2018). Recently, promising advances in anodizing to form a compact topology of memristors (8–29 nm thickness and 4 μm^2^ feature area) have been demonstrated (Aglieri et al., 2018). The method is relatively cheap and is typically performed at ambient temperature. However, precise control of film thickness and sensitivity to the type and surface of the substrate are major challenges.

##### Atomic layer deposition (ALD)

Chemical vapor-based deposition techniques are widely used in the fabrication of thin films. Ultrathin TiO_2_ layers in memristive devices are usually fabricated by ALD or plasma-enhanced ALD (PEALD). In these methods, Ti-based precursors (TiCl_4_, titanium alkoxides) are decomposed in the presence of an oxidizer (H_2_O, O_3_, or O_2_) (Seo et al., 2011; Marichy et al., 2012). The PEALD process can be performed at relatively low substrate temperatures (Kwon et al., 2010). The methods allow very thin TiO_2_ layers in the range of 7–13 nm to be formed (Jeong et al., 2010a,b) and are compatible with other fabrication techniques. Using a combination of ALD and other nanofabrication processes, a very small topological feature size of 4 nm^2^ has been achieved for memristor crossbar arrays (Pi et al., 2019). The ALD methods are relatively costly, but provide high precision and thus scalability and reproducibility.

#### Physical Approaches

##### Physical vapor deposition (PVD)

These methods require the transfer and deposition of materials under vacuum. The post-processing annealing at 200–600°C may support adhesion and crystallization (Cortese et al., 2016; Haidry et al., 2017). The sputtered TiO_2_ layers meet the criteria of the nanometer-range electronics industry (Strachan et al., 2013; Ghenzi and Levy, 2018) and therefore find application at an industrial level, especially in reproducible, long-lasting, and portable RRAM devices (Nickel et al., 2013). The methods are sensitive to contamination inside the chamber and require high power (Acharyya et al., 2014).

##### Pulsed microplasma cluster source (PMCS)

This technique forms supersonic pulsed beams of the metal oxide clusters and deposits them on a substrate. Deposition occurs at high energy and results in nanostructured thin films. The method was suggested for fabrication of TiO_2_ memristors, as the nanocrystalline structure and porosity can be controlled by varying deposition parameters, while the growth can be performed at room temperature (Baldi et al., 2015). In addition to purely memristive applications, the method was also suggested for fabrication of biohybrid TiO_2_ interfaces (Roncador et al., 2017) that demonstrated good properties for the growth and vitality of neuronal cell cultures and their electrical activity.

### Fabrication

Thin-film fabrication implies depositing TiO_2_ nanomaterials onto a substrate along with arrangement of the electrodes. In this section we briefly outline the most widely used techniques for fabrication of TiO_2_ memristive devices.

#### Drop Casting

This is the simplest technology. Functional layers are formed by depositing drops of colloidal dispersions of TiO_2_ onto a substrate using a syringe or pipette. The thickness of the TiO_2_ layers obtained by this method typically ranges between 40 and 200 μm (Gale et al., 2014; Abunahla et al., 2018; De Carvalho et al., 2019). This thickness range does not match the usual topological features of RRAM memristors, although the method has recently been justified for various memristive sensors (Abunahla et al., 2016, 2018; Sahu and Jammalamadaka, 2019).

#### Spin Coating

This method is based on spinning of the substrate, which exploits an inertial force acting on the fixed substrate and an unfixed drop of slurry cast on top of it. Varying the viscosity of the slurry or solution and the rotation speed, the method may be adjusted to obtain TiO_2_ thin films with thicknesses down to 35 nm (Tao et al., 2020). Thus, the method has been proposed for fabrication of RRAM (Hu et al., 2020).

#### Dip Coating

Dip coating is considered to be a high-quality and cost-efficient deposition method, if physical adsorption between the substrate and the adsorbate dispersed in a colloidal solution is adopted as a controllable process. In this way, memristive thin films comprising two-dimensional TiO_2_ flakes with a thickness of ~2 nm were obtained by dip coating. These films demonstrated distinctive properties such as a high resistive switching ratio, fast switching speed, and extremely low erase energy consumption (Dai et al., 2017).

#### Inkjet Printing

Inkjet printing is a cost-efficient fabrication method, especially for micro- and nanopatterning and laboratory-scale prototyping (Menard et al., 2007). The method has been applied for stretchable and flexible electronics (Nayak et al., 2019), including various TiO_2_ memristive devices (Samardzić et al., 2015). Inkjet printers are used for automated drop casting and typically operate with a picoliter droplet volume and provide droplet deposition with 10–20 μm spatial resolution. The thickness of the film may be variable and usually exceeds 100 nm (Duraisamy et al., 2012), although a lower value of 80 nm has been reported recently (Salonikidou et al., 2019).

### Annealing and Electric Properties

Annealing in reducing atmospheres affects the concentration of charged point defects and thus the resistive switching. Despite many experimental studies, including the post-fabrication thermal annealing stage (typically at 400–600°C) under vacuum (Schmidt et al., 2015), nitrogen (Seo et al., 2011; Cortese et al., 2016; Regoutz et al., 2016), argon (Nelo et al., 2013), or N_2_ + H_2_ (4–5%) gas mixtures (Yang et al., 2008; Miller et al., 2010), only a few studies have systematically addressed the effects of thermal treatment under various annealing atmospheres on the resistive switching behavior of TiO_2_ memristive devices (Lai et al., 2013a,b; Nelo et al., 2013).

## Summary and Outlook

The current renaissance of the resistive switching phenomenon over the last 12 years has been intimately associated with studies on TiO_2_ thin films that have often been addressed as prototype oxide memristors for many research applications. Numerous recent achievements highlighted in this mini-review demonstrate the rapid development of TiO_2_-based memristors in various application fields, while the growing interest in these devices is seemingly far from saturation.

The major challenges of TiO_2_ memristors have been outlined in several previous reviews and remain essentially unsolved. Technologically, the emerging properties of memristive systems concern the operational stochasticity, number of distinguishable states, switching energy, switching speed, endurance, retention, and feature size. Their relationship in various types of modern memristive systems has been comprehensively addressed in a recent topical review (Zhang et al., 2020). Besides the permanent technological issues of increasing the integration density and reducing the production costs, there are fundamental challenges in understanding the mechanisms of resistive switching in solids, which, in practice, limit the scalability and reproducibility of the memristive devices (Acharyya et al., 2014; Jeong et al., 2016; Zidan, 2018; Xia and Yang, 2019; Zhang et al., 2020). Despite the apparent simplicity of the metal–insulator–metal configuration, the mechanisms involved in the memristive electric performance are manifold and complex. Two types of electroforming processes, electronic (Shao et al., 2015) and ionic (Waser et al., 2009), play essential roles in the non-linearity and hysteresis of the voltage–current relationship. Thus, understanding the electron band structure of TiO_2_ polymorphs (Scanlon et al., 2013), the defect structure (Bak et al., 2006), and the equilibrium relations with Magnéli phases (Padilha et al., 2016) are of key importance and should help to address the challenges at a theoretical level. In addition, many experimental studies have recently addressed tuning of the electric properties of TiO_2_ memristors by choosing appropriate electrodes and an appropriate operating voltage regime or by affecting the phase ratio, defect structure, and microstructure of the synthesized materials using post-processing annealing under ambient or inert atmospheres (Goren et al., 2014; Schmidt et al., 2015; Cortese et al., 2016; Regoutz et al., 2016; Haidry et al., 2017; Tao et al., 2020). Thus, they have contributed to our understanding of the complex resistive switching phenomena in TiO_2_. Meanwhile, only a few studies have systematically addressed the effect of thermal annealing at reduced oxygen partial pressures on the resistive and resistive switching properties of TiO_2_ thin films (Lai et al., 2013a,b; Nelo et al., 2013). Seemingly, this issue also remains underexplored.

In view of the current trends and challenges of TiO_2_-based memristors, we can expect an increasingly large role of chemical approaches to device fabrication, lowering of the production costs, rapid development of neuromorphic computing systems, and further convergence of artificial electronic neurons with biological cells based on TiO_2_ thin films.

## Author Contributions

All authors listed have made a substantial, direct and intellectual contribution to the work, and approved it for publication.

## Conflict of Interest

The authors declare that the research was conducted in the absence of any commercial or financial relationships that could be construed as a potential conflict of interest.
